# 

**Published:** 1931

**Authors:** 


### CONTENTS.

| | Page |
|---|---|
| The Long Fox Memorial Lecture: The Partnership between Anæsthesia and Surgery. By Arthur L. Flemming, M.B., Ch.B., L.R.C.P., M.R.C.S. | 233 |
| Silicosis: I.—Administrative and Clinical. By J. A. Nixon, C.M.G., M.D., F.R.C.P. | 253 |
| Silicosis: II.—Radiology and Pathology. By G. B. Bush, M.B., C.B., D.M.R.E. | 259 |
| Congenital Abnormalities of the External Ear. By E. Watson-Williams, M.C. | 273 |
| Hæmaturia in the Pre-Eruptive Stage of Measles. By F. G. Jenkins, M.B., Ch.B. | 275 |
| Cyanosis in the Fatal Broncho-Pneumonia of Influenza. By Frank E. Fletcher | 277 |
| Reviews of Books | 283 |
| Editorial Notes | 289 |
| Meetings of Societies | 292 |
| The Medical Library of the University of Bristol | 296 |
| Local Medical Notes | 299 |

